# Supplementary material for: Stress-induced nuclear translocation of ONAC023 improves drought and heat tolerance through multiple processes in rice
Source: Nat Commun. 2024 Jul 13;15:5877. doi: 10.1038/s41467-024-50229-9 (PMC11245485; doi:10.1038/s41467-024-50229-9)
Supplement: Supplementary file 1 — Supplemental Information [file 41467_2024_50229_MOESM1_ESM.pdf]

Supplementary Figures

a

Tissue	Variety	Treatment	Uniquely mapped reads	Replicate
Panicle (flowering stage)	ZH11	Heat (45°C for 0.5 h)	79,742,374	1
Panicle (flowering stage)	ZH11	Heat (45°C for 0.5 h)	84,297,080	2
Panicle (flowering stage)	ZH11	Normal (30°C)	62,819,220	1
Panicle (flowering stage)	ZH11	Normal (30°C)	65,187,928	2
Seedling (4-leaf stage)	ZH11	Drought (RSM < 10% for 3 d)	78,104,230	1
Seedling (4-leaf stage)	ZH11	Drought (RSM < 10% for 3 d)	92,329,142	2
Seedling (4-leaf stage)	ZH11	Drought (RSM < 10% for 3 d)	65,288,398	3
Seedling (4-leaf stage)	ZH11	Heat (45°C for 10 h)	68,875,418	1
Seedling (4-leaf stage)	ZH11	Heat (45°C for 10 h)	63,101,346	2
Seedling (4-leaf stage)	ZH11	Heat (45°C for 10 h)	58,092,960	3
Seedling (4-leaf stage)	ZH11	Normal (RSM = 100%, 26°C)	69,041,382	1
Seedling (4-leaf stage)	ZH11	Normal (RSM = 100%, 26°C)	55,507,960	2
Seedling (4-leaf stage)	ZH11	Normal (RSM = 100%, 26°C)	72,219,146	3
Seedling (4-leaf stage)	DJ	Drought (RSM < 10% for 5 d)	68,022,456	1
Seedling (4-leaf stage)	DJ	Drought (RSM < 10% for 5 d)	59,825,706	2
Seedling (4-leaf stage)	DJ	Drought (RSM < 10% for 5 d)	61,654,286	3
Seedling (4-leaf stage)	DJ	Normal (RSM = 100%, 26°C)	62,391,162	1
Seedling (4-leaf stage)	DJ	Normal (RSM = 100%, 26°C)	84,062,976	2
Seedling (4-leaf stage)	DJ	Normal (RSM = 100%, 26°C)	65,984,044	3

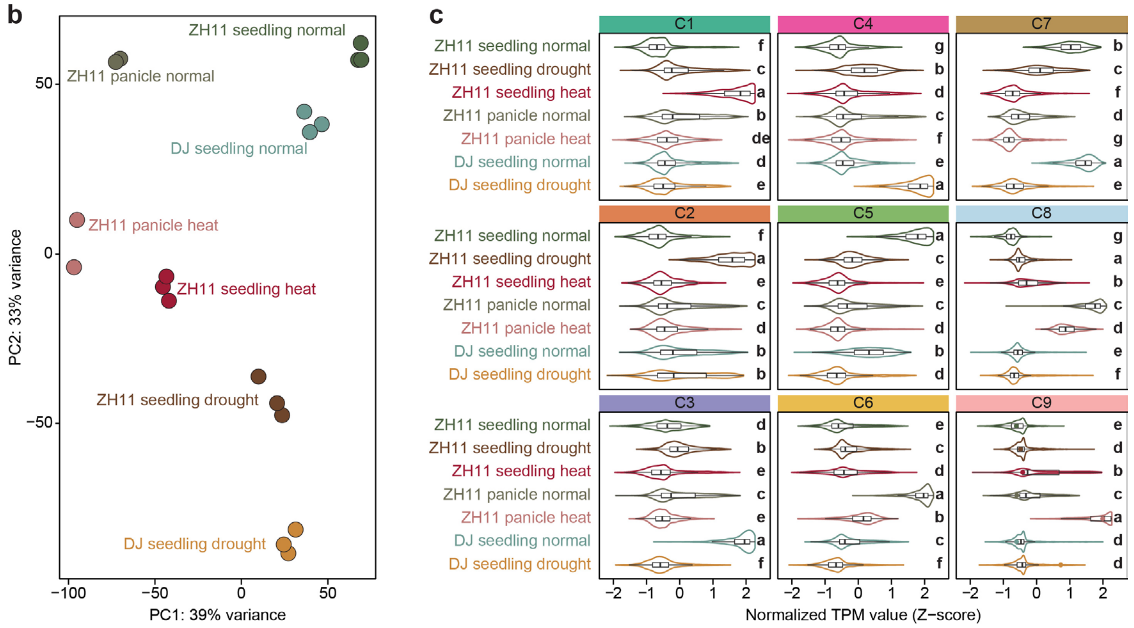

Supplementary Fig. 1: Supplemental information related to the NAC-centered GRN analysis (supports Fig. 1).

a List of the RNA-seq samples used in the NAC-centered GRN construction. **b** Principal component analysis (PCA) showing the reproducibility of the RNA-seq samples. **c** Violin plots showing the Z-transformed TPM values of the DEGs in each ICA cluster. Letter symbols denote significances ($P < 0.05$) determined by one-way ANOVA (two-sided) with Tukey's Honest significant difference (HSD) test. Exact P-values and details of the one-way ANOVA–HSD test can be found in the Source Data file. Source data are provided as a Source Data file.

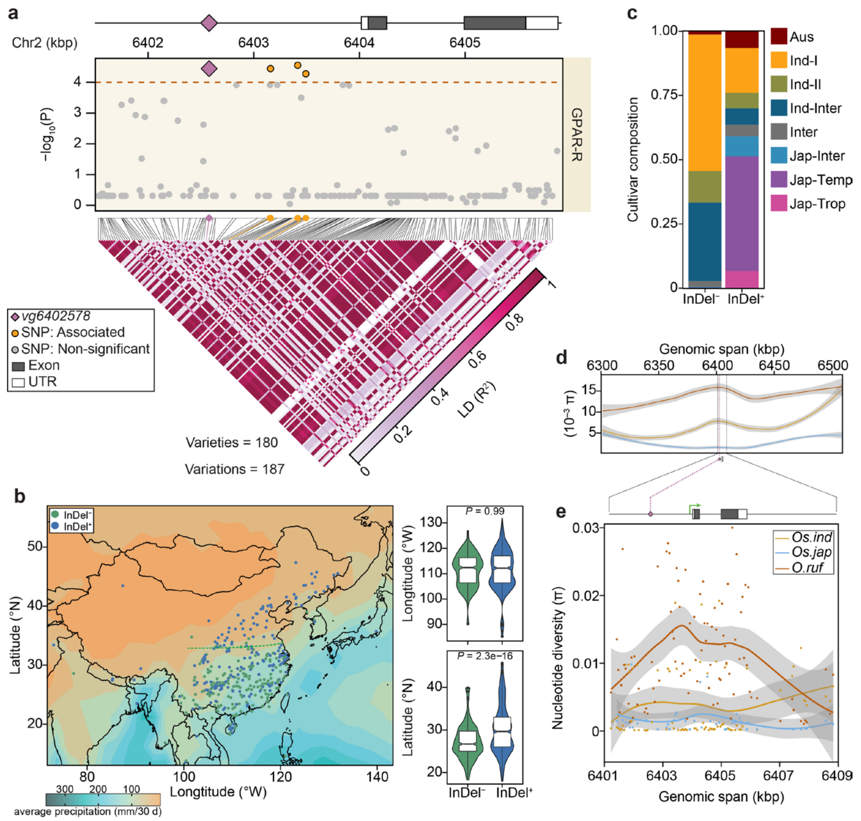

Supplementary Fig. 2: Natural variation and nucleotide diversity of *ONAC023* (supports Fig. 1). **a** Enlarged view of the GWAS result in Fig. 1c. **b** Geographic distribution of the accessions with available information within the realm of East Asia. The green dashed line indicates the approximate boundary between the north and south climate zones in China. Latitude and longitude differences between the InDel$^+$ and InDel$^-$ groups were shown by the violin plots and tested by two-tailed Student's *t*-test. Color zones in the map indicate the monthly average rainfall values (from Jan,1976 to Jun,2021) collected by NOAA. **c** Rice subgroup compositions of the InDel$^-$ and InDel$^+$ varieties. Ind, *Os. Indica*; Jap, *Os. japonica*; Inter, intermedia; Temp, temperate; Trop, tropical. **d** Nucleotide diversity analysis within the ±100 kbp region of the *ONAC023* locus. The changing trends of the 200-bp windowed π values are illustrated by LOESS regression. *Os. ind*, *Os. indica*; *Os. jap*, *Os. japonica*; *O. ruf*, *O. rufipogon*. **e** Profile of the nucleotide diversities among *Os. indica* (*Os. ind*), *Os. japonica* (*Os. jap*), and *O. rufipogon* (*O. ruf*) in the ±3 kb region of the *ONAC023* gene locus. Data points represent the π values in a 200-bp window and the changing trend of the π value within the region was illustrated by LOESS regression. The blue quadrangles in **(d)** and **(e)** indicate the site of InDel *vg2402578*. Source data are provided as a Source Data file.

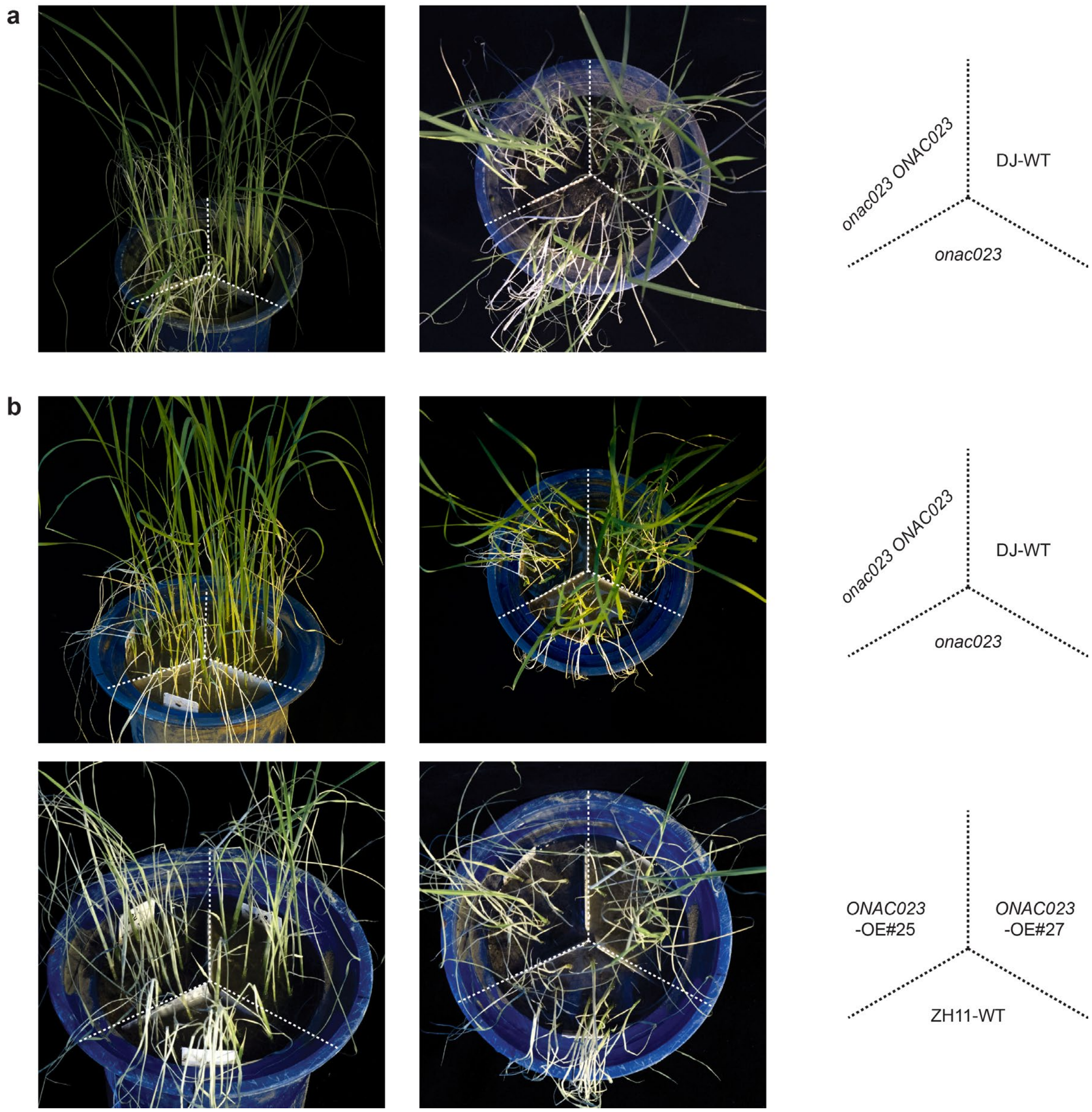

Supplementary Fig. 3: Raw photos for the color-processed images in Figs. 2 and 3 (supports Figs. 2 and 3).

a–**b** Raw photos for the corresponding seedling images in Fig. 2a (**a**) and Fig. 3a (**b**), respectively.

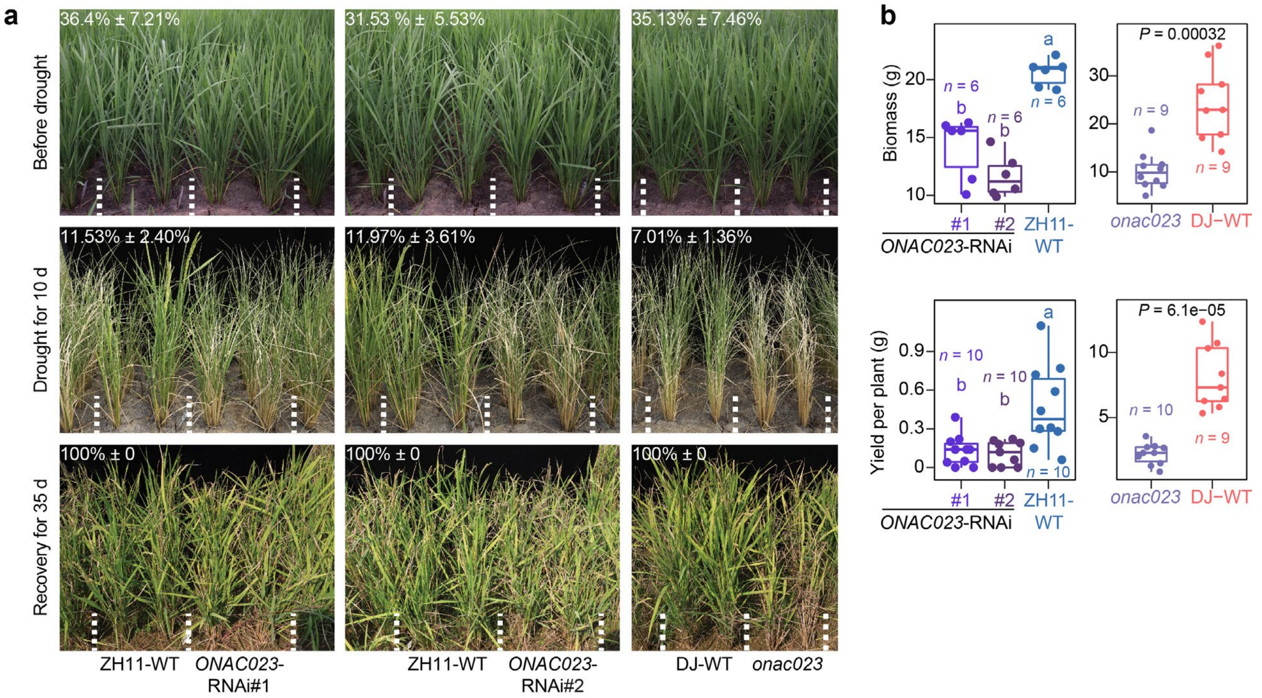

Supplementary Fig. 4: *ONAC023* RNA-interfering (*ONAC023*-RNAi) and *onac023* T-DNA mutants were sensitive to drought stress (supports Fig. 2).

a Phenotype comparison of *ONAC023*-RNAi lines and *onac023* to the corresponding WT plants under in-field drought stress treatment. The RSMs were expressed as mean ± SD based on three randomized blocks. One representative of the three randomized blocks is shown. **b** Yield (per plant) and biomass statistics of the drought-treated plants after recovery. One-way ANOVA–HSD test (two-sided, at $P < 0.05$) was applied to determine the statistical significance based on 6–10 plants from the three randomized blocks. Exact *P*-values and details of the one-way ANOVA–HSD test can be found in the Source Data file. Source data are provided as a Source Data file.

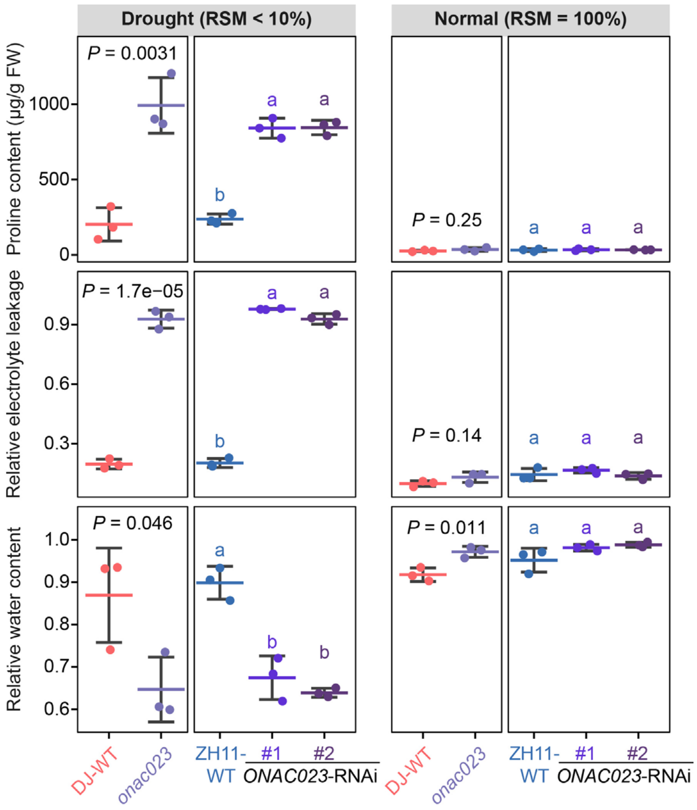

Supplementary Fig. 5: Physiological traits measurement for *onac023*, ONAC023-RNAi, and the corresponding CKs (supports Fig. 2).

Cross-bars indicate mean ± SD of three repeats of the assay (at least 10 plants were sampled for each replicate). Two-tailed Student's *t*-test was applied to determine the differences. FW, fresh weight of the sampling tissue. Source data are provided as a Source Data file.

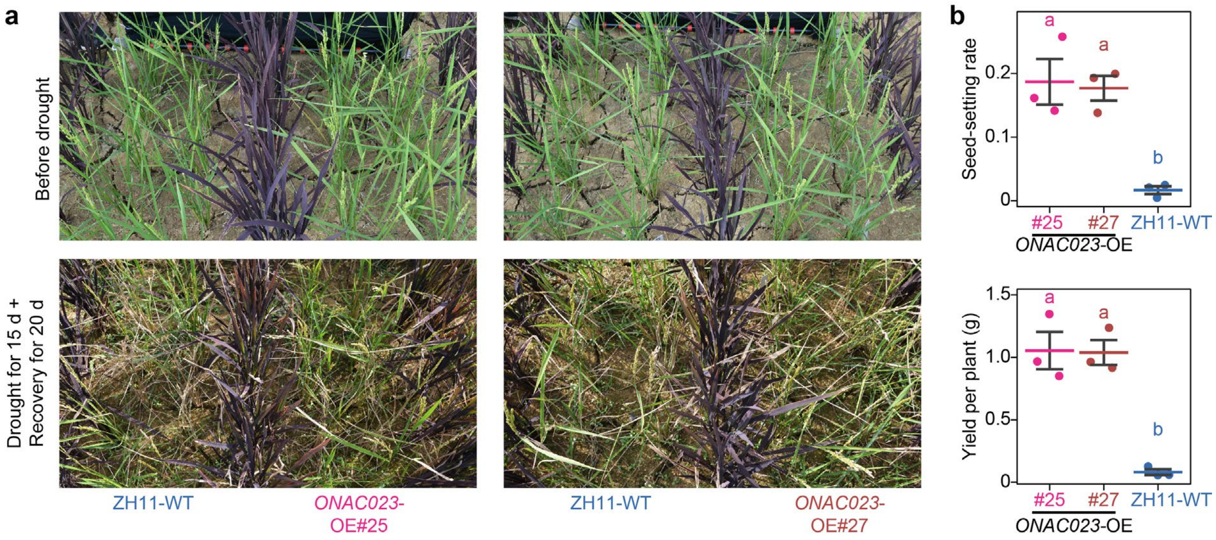

Supplementary Fig. 6: Drought tolerance evaluation on the *ONAC023*-OE plants in Hainan province (supports Fig. 2).

a In-field drought tolerance evaluation of the *ONAC023* overexpressors in Hainan province. Phenotype of one out of three repeats is shown for each transgenic line. **b** Statistics on the seed-setting rate and yield (per plant) of the tested blocks. Cross-bars indicate mean ± SEM of three randomized blocks. Letter symbols denote significances ($P < 0.05$) determined by one-way ANOVA–HSD test (two-sided). Exact P-values and details of the one-way ANOVA–HSD test can be found in the Source Data file. Source data are provided as a Source Data file.

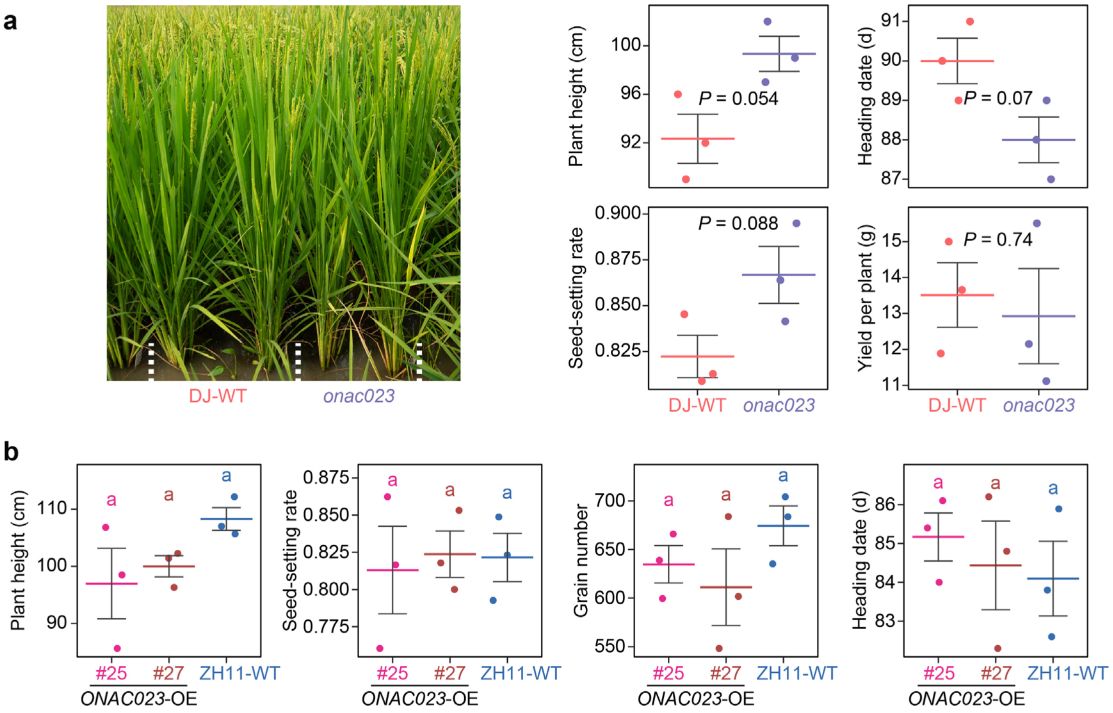

Supplementary Fig. 7: The agronomic traits of *onac023* and *ONAC023*-overexpressing lines under the normal field condition (supports Fig. 2).

a Phenotype of the *onac023* and DJ-WT under the normal condition. **b** Trait statistics of the *ONAC023* overexpressors under the normal condition. Cross-bars denote mean ± SE based on three randomized blocks. Letter symbols denote significances ($P < 0.05$) determined by one-way ANOVA–HSD test (two-sided). Exact *P*-values and details of the one-way ANOVA–HSD test can be found in the Source Data file. Source data are provided as a Source Data file.

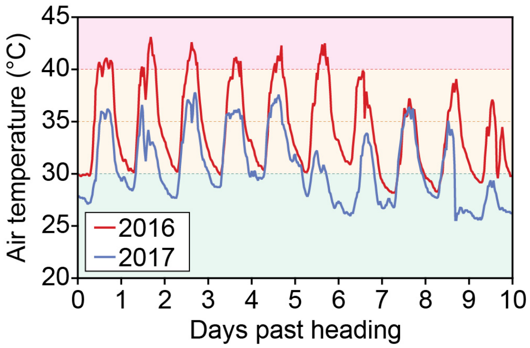

Supplementary Fig. 8: Records of the air temperature during the panicle heading process of the investigated plants in 2016 and 2017 (supports Fig. 3).

The *X*-axis zero-point indicates the starting date of panicle heading. Source data are provided as a Source Data file.

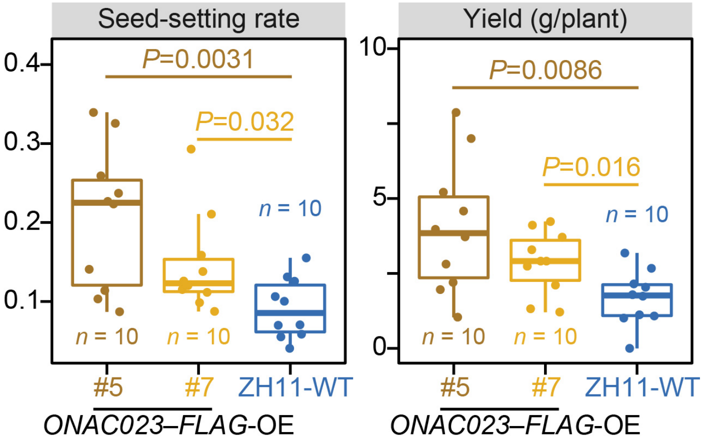

Supplementary Fig. 9: Statistics of seed-setting rate and yield (per plant) of the *ONAC023–FLAG*-OE transgenic plants compared with ZH11-WT after recovery from the heat stress under field conditions (supports Fig. 3).

The traits were evaluated in the year of 2016. Two-tailed Student's *t*-test was applied to determine the statistical significance (based on 10 plants from three randomized blocks). *n*, number of plants. Source data are provided as a Source Data file.

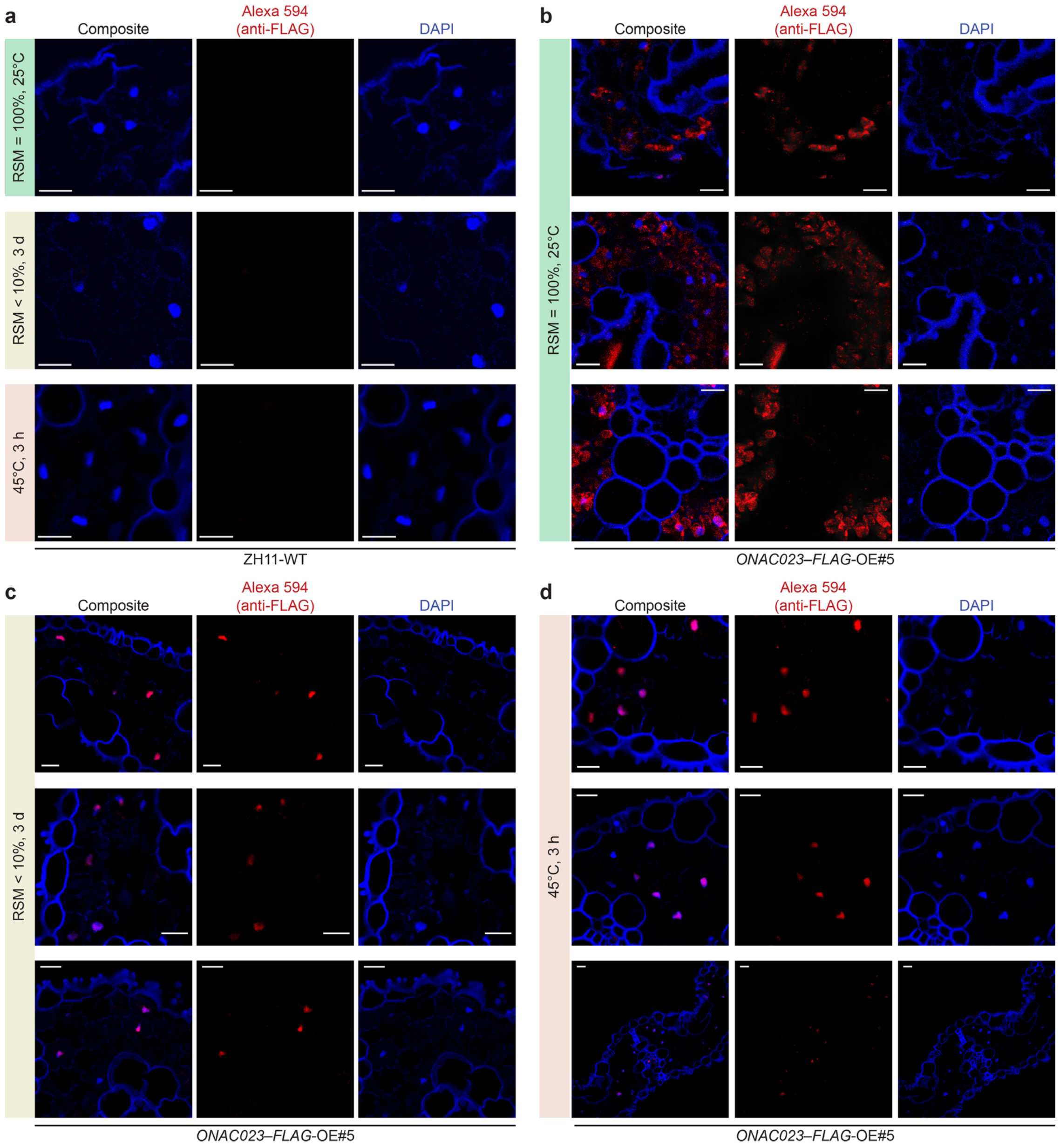

Supplementary Fig. 10: Immunofluorescence assay results on ZH11-WT and additional replicates of *ONAC023–FLAG*-OE#5 (supports Fig. 4).

a Control immunofluorescence slices on ZH11-WT under normal (RSM = 100%, 25°C), drought (RSM < 10% for 3 d), and heat (45°C for 3 h) conditions. **b–d** Immunofluorescence slices on additional replicates of *ONAC023–FLAG*-OE#5 under normal (**a**), drought (**b**), and heat (**c**) conditions. Each row illustrates the multi-channel views on a slice from the seedling in one of the three repeats of the treatments.

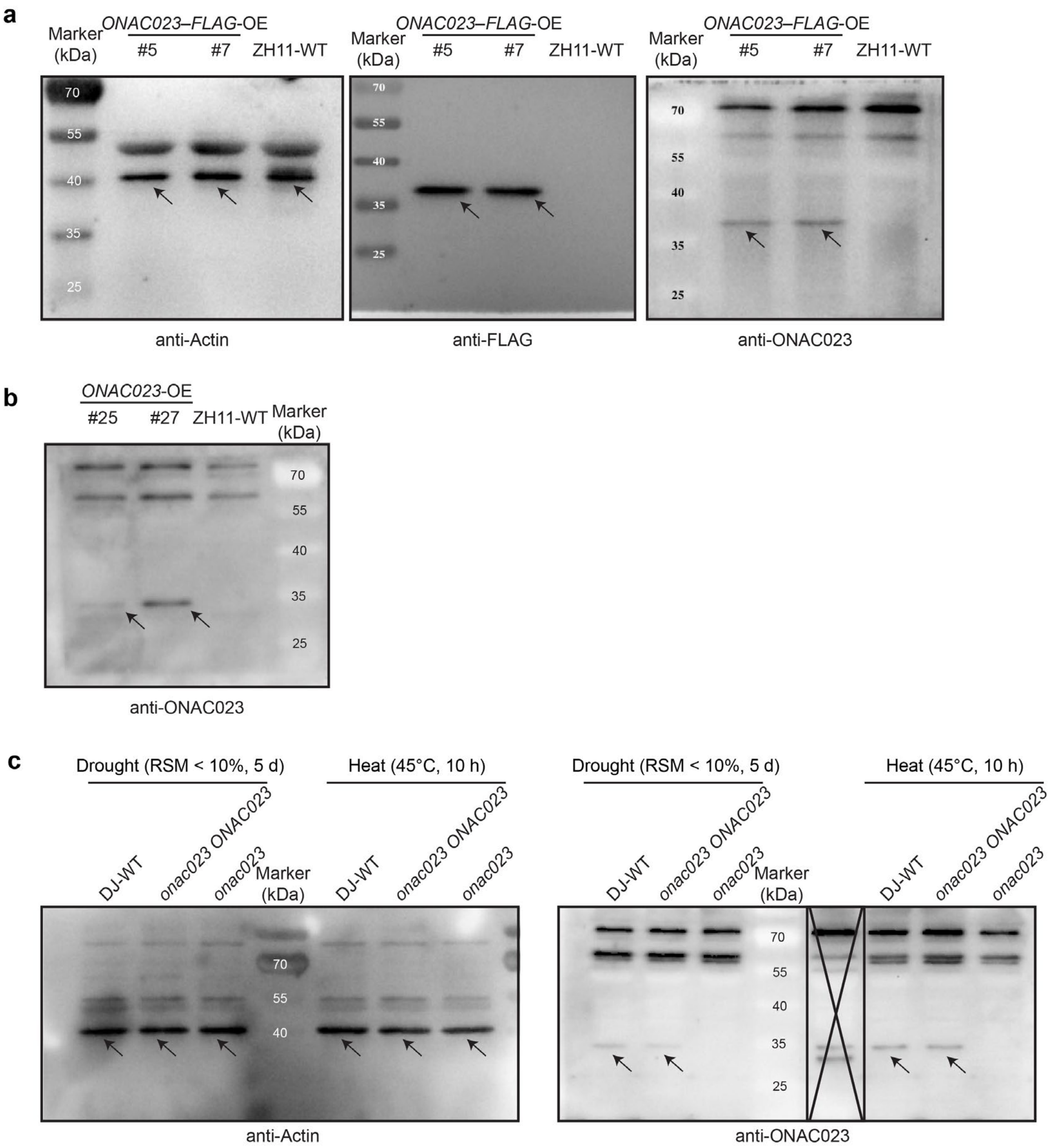

Supplementary Fig. 11: Validation of the ONAC023-specific polyclonal antibody (supports Figs. 4 and 8).

a–**c** Western blots were performed with the total proteins extracted from the 4-leaf seedlings grown under normal condition (**a** and **b**) and drought or heat stress condition (**c**). Bands specific to the antibody are indicated by arrows. The lane irrelevant to this study is marked by cross. All experiments were repeated three times and a representative image is shown. Source data are provided as a Source Data file.

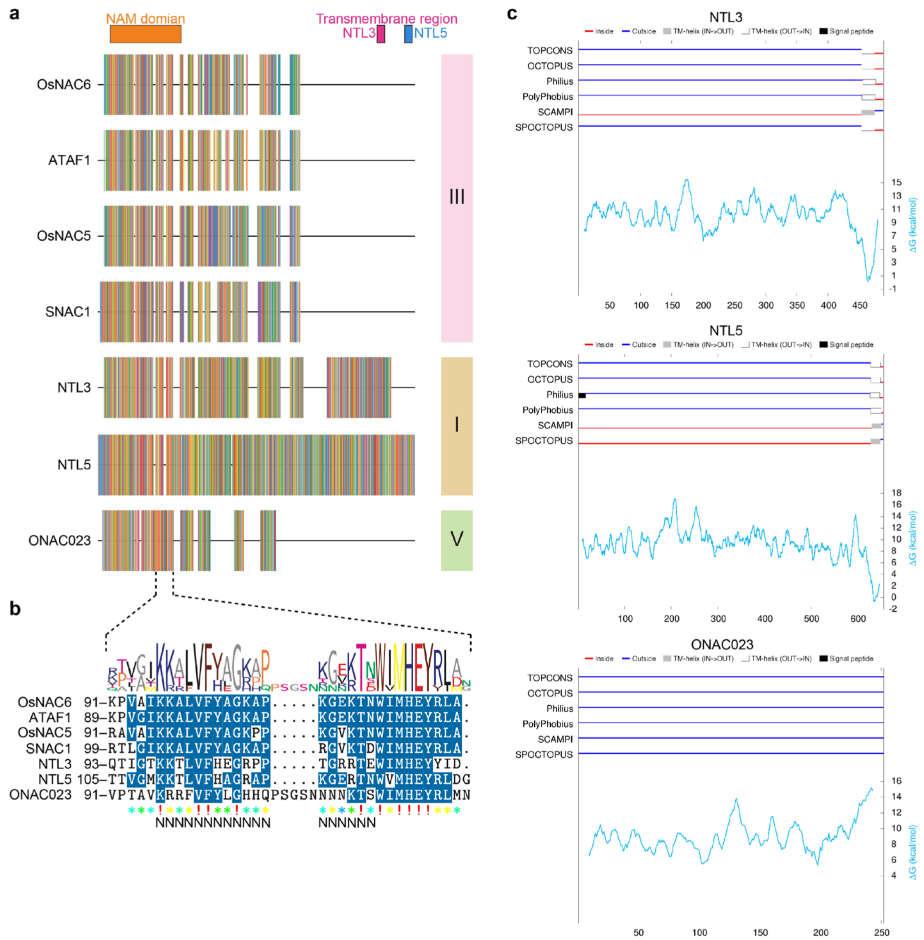

Supplementary Fig. 12: Protein sequence comparisons between ONAC023 and NACs with transmembrane domain (supports Fig. 4).

a Protein sequence alignment of ONAC023 to the transmembrane domain-containing NACs in rice (NTL3 [LOC_Os01g15640] and NTL5 [LOC_Os08g44820]) as well as the representatives of stress-responsive NACs in rice (OsNAC5 [LOC_Os11g08210], OsNAC6 [LOC_Os01g66120], and SNAC1) and *Arabidopsis* (ATAF1 [AT1G01720]). Positions corresponding to NAM domain and predicted transmembrane regions in NTL3 and NTL5 are marked by rectangles. **b** Snapshot of the aligned region containing the conserved NAC nuclear localization signal. Residuals with predicted nuclear localization signaling function are marked with “N”. **c** Transmembrane region predictions of ONAC023, NTL3, and NTL5 by TOPCONS. Predicted transmembrane regions were marked with white or grey rectangles. No transmembrane region was detected in ONAC023.

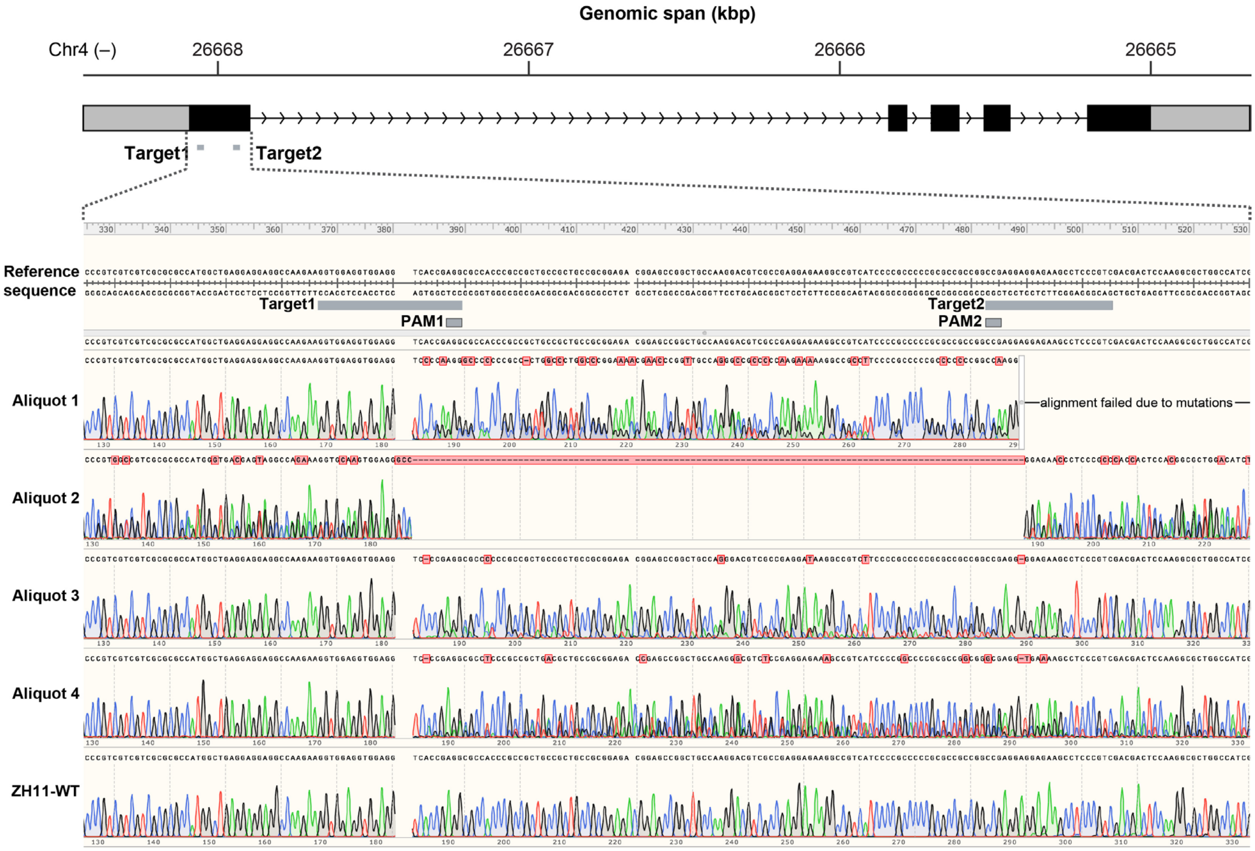

Supplementary Fig. 13: Genotypic verification of CRISPR-Cas9-mediated *OsREM1.5* mutations in ZH11 protoplasts (supports Fig. 5).

Four aliquots of the *osrem1.5* cells in Fig. 5e were sequenced. WT represents ZH11-WT protoplasts transfected with blank pRGEB32 vector. The mutated bases are highlighted by red rectangles.

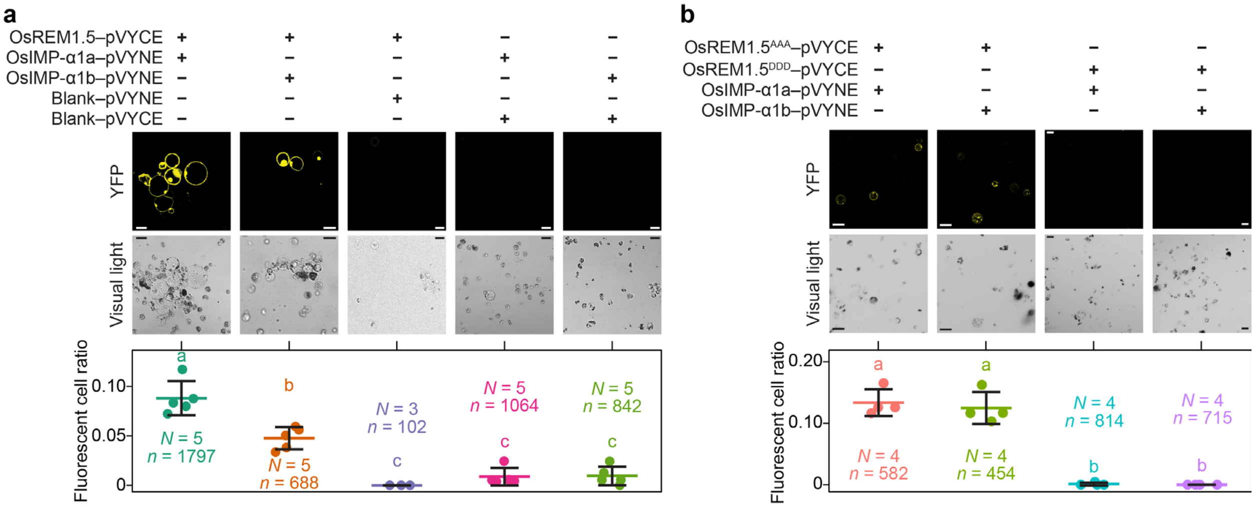

Supplementary Fig. 14: Validation of the interactions between OsREM1.5 and the importin-α proteins by BiFC (supports Figs. 5 and 7).

a–**b** Microscopic images of the OsREM1.5–OsIMP-α BiFC slices with OsREM1.5-WT (**a**) and OsREM1.5 phosphomutants (**b**). The cross-bar plots show the statistics of the BiFC fluorescent cell ratio (mean ± SD by four or five repeats of the assay). *N*, number of repeats of the assay. *n*, number of observed cells. Letter symbols denote significances ($P < 0.05$) determined by one-way ANOVA–HSD test (two-sided). Exact *P*-values and details of the one-way ANOVA–HSD test can be found in the Source Data file. Source data are provided as a Source Data file.

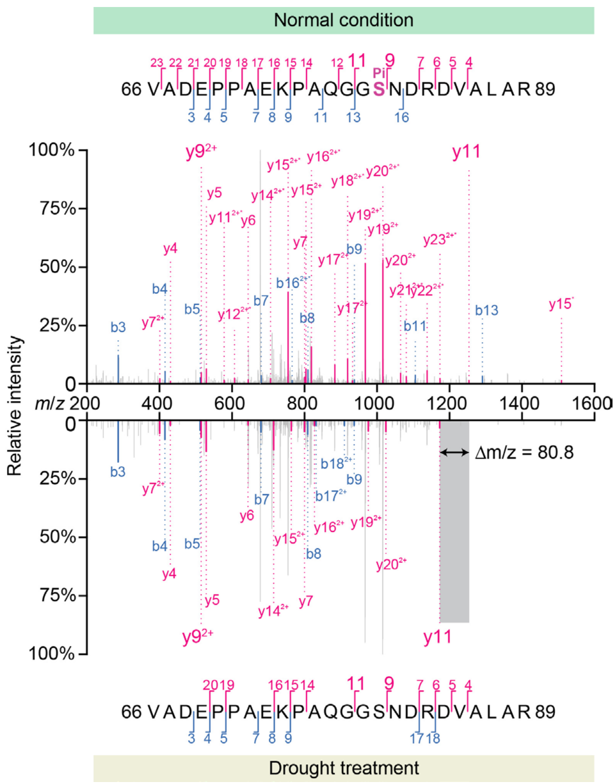

Supplementary Fig. 15: The MS2 spectrum plot showing the differential phosphorylation of OsREM1.5 S80 under the normal and drought stress conditions in cultivar HY73 (supports Fig. 7). Δ*m*/*z* caused by the dephosphorylation of S80 under drought treatment was denoted with grey rectangle.

a

Sample	Seq length (bp)	Layout	Properly paried reads	Uniquely mapped, non-redundant reads	Effective Depth (× genome) *	Effective peaks **
DJ-WT Drought IP rep1	150	paired	97,446,346	70,559,816	27.80	2,140
DJ-WT Drought IP rep2	150	paired	95,184,532	68,301,588	26.99	
DJ-WT Heat IP rep1	150	paired	97,062,966	69,206,228	27.54	22,056
DJ-WT Heat IP rep2	150	paired	93,465,498	75,250,796	29.93	
DJ-WT Heat IP rep3	150	paired	92,725,688	74,263,564	29.38	
onac023 Drought IP rep1	150	paired	83,073,698	60,121,146	23.81	#N/A
onac023 Drought IP rep2	150	paired	82,553,488	59,133,938	23.39	
onac023 Heat IP rep1	150	paired	70,029,852	53,091,738	21.26	
onac023 Heat IP rep2	150	paired	81,238,844	61,537,314	24.65	
onac023 Heat IP rep3	150	paired	83,394,962	61,547,144	24.65	
DJ-WT input	150	paired	91,253,818	72,966,366	29.25	
onac023 input	150	paired	78,197,734	58,154,692	23.29	
ONAC023–FLAG-OE#5 Normal IP rep1	150	paired	72,018,236	8,646,770	3.44	
ONAC023–FLAG-OE#5 Normal IP rep2	150	paired	72,402,018	8,986,272	3.58	
ZH11-WT Normal IP rep1	150	paired	93,694,812	2,917,046	0.76	
ZH11-WT Normal IP rep2	150	paired	71,929,798	1,923,754	1.16	
ONAC023–FLAG-OE#5 Normal input	150	paired	86,303,154	69,328,494	26.96	
ZH11-WT Normal input	150	paired	65,228,786	53,961,862	20.99	

*: Sequencing depth by uniquely mapped, non-redundant bases.

**: Peaks specific to ONAC023 binding in target gene promoter regions. #N/A, not applicable.

b

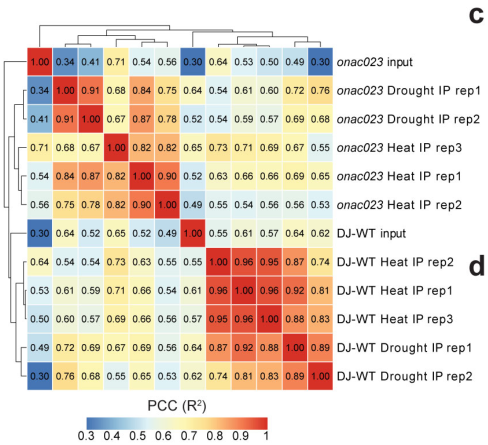

c

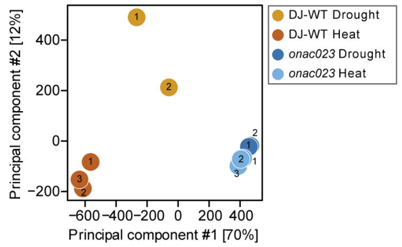

d

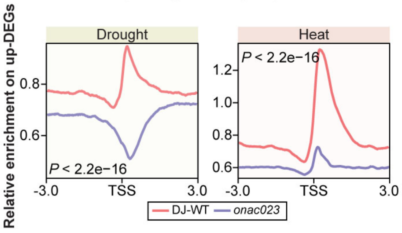

e

Sample	Read length (bp)	Layout	Properly paried reads	Uniquely mapped reads	Effective depth (× genome)	Expressed genes (TPM > 1) *
DJ-WT Drought rep1	150	paired	88,070,792	84,823,466	33.76	20,586
DJ-WT Drought rep2	150	paired	82,218,466	77,868,644	30.83	21,374
DJ-WT Drought rep3	150	paired	79,682,498	76,832,720	30.34	21,301
DJ-WT Heat rep1	150	paired	81,061,388	76,849,890	30.57	19,331
DJ-WT Heat rep2	150	paired	92,048,942	86,328,672	34.35	18,959
DJ-WT Heat rep3	150	paired	73,934,158	69,980,614	27.85	19,560
onac023 Drought rep1	150	paired	72,207,384	67,915,896	27.05	19,011
onac023 Drought rep2	150	paired	66,250,414	62,749,620	24.87	18,682
onac023 Drought rep3	150	paired	67,020,644	64,503,870	25.68	18,829
onac023 Heat rep1	150	paired	91,101,568	87,055,574	34.66	17,398
onac023 Heat rep2	150	paired	88,782,874	82,845,016	32.93	17,484
onac023 Heat rep3	150	paired	101,515,462	96,399,500	38.40	17,332

*: Gene models were according to MSU v7.0

f

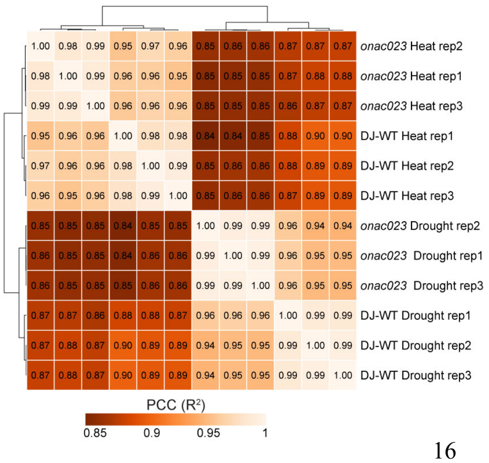

g

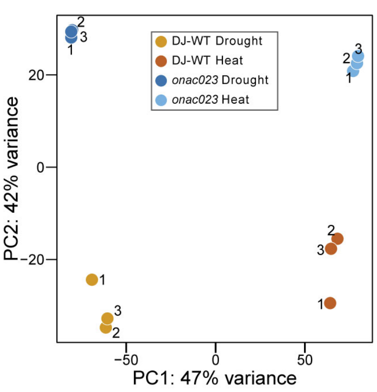

Supplementary Fig. 16: Quality control profiles for the RNA-seq and ChIP–seq (supports Fig. 8). **a** Summary of the ChIP–seq libraries. **b** Heatmap showing the PCC (R^2) of the ChIP–seq result. **c** PCA based on the quantified peaks of the ChIP–seq samples. **d** DJ-WT and *onac023* ChIP–seq profiles (expressed as IP vs input) at the TSS regions of the up-regulated DEGs in DJ-WT compared to *onac023*. *P*-values were determined by two-tailed Student's *t*-test. **e** Summary of the sequence data from RNA-seq libraries. **f** Heatmap showing the PCC (R^2) of the RNA-seq result. **g** PCA of the RNA-seq samples.

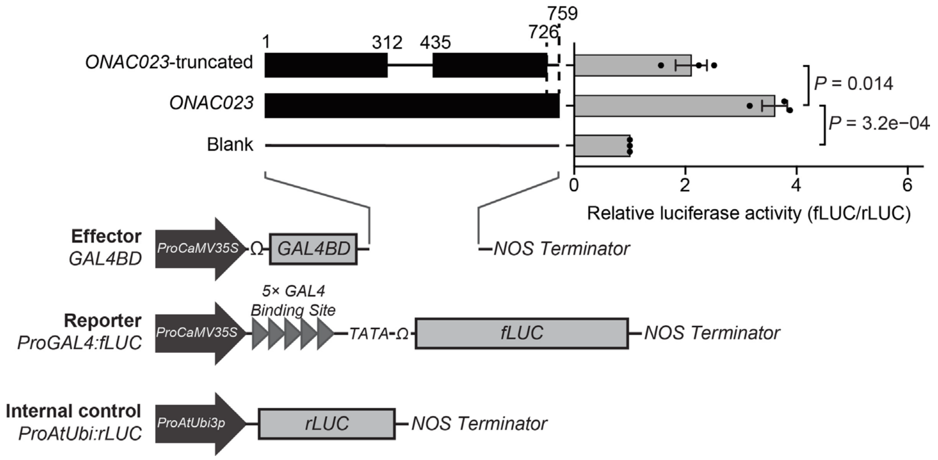

Supplementary Fig. 17: Transcriptional activity of ONAC023 by dual-LUC assay (supports Fig. 8).

The histogram indicates the mean relative luciferase activity (normalized to the "Blank" construct). Error bars represent the SEM of three repeats of the assay (each included three independent transfections). Statistic significances were determined by two-tailed Student's *t*-test. Source data are provided as a Source Data file.

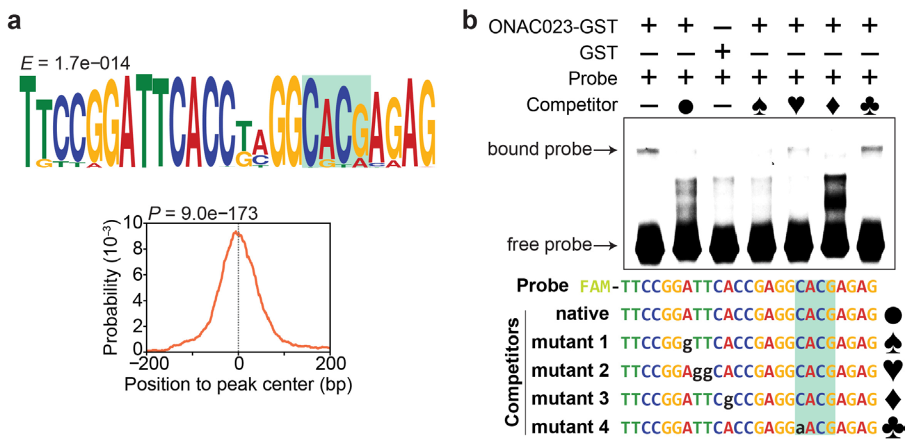

Supplementary Fig. 18: Validation of the consensus ONAC023-binding sequence by EMSA (supports Fig. 8).

a Consensus ONAC023-binding sequence determined by MEME. Distribution probability of the sequence in relation to the ChIP–seq peak center is shown by the line plot. *P*-value of the central enrichment was determined by binomial test (two-sided). **b** EMSA validation of ONAC023-binding to the consensus sequence. Mutated bases in the competitor probes are expressed in lower case. Source data are provided as a Source Data file.

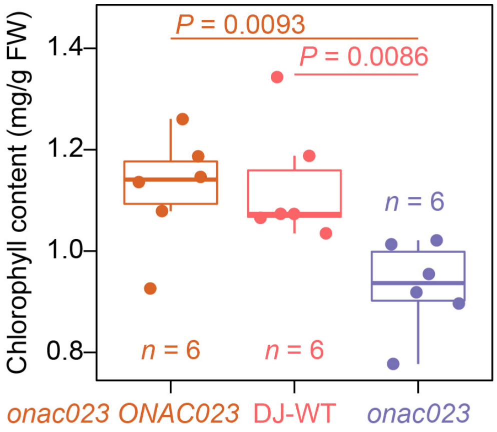

Supplementary Fig. 19: Chlorophyll contents of DJ-WT and *onac023* under drought stress (supports Fig. 8).

Two-tailed Student's *t*-test was applied to compare the differences between the average values. Dots denote data from independent plants (n = 6). The lower, central, and upper hinges of the box plots correspond to the 25th, 50th (median), and 75th percentiles, respectively. The whiskers extend from the hinges to the largest/smallest values no further than 1.5× IQR (inter-quartile range) from the hinges. Source data are provided as a Source Data file.

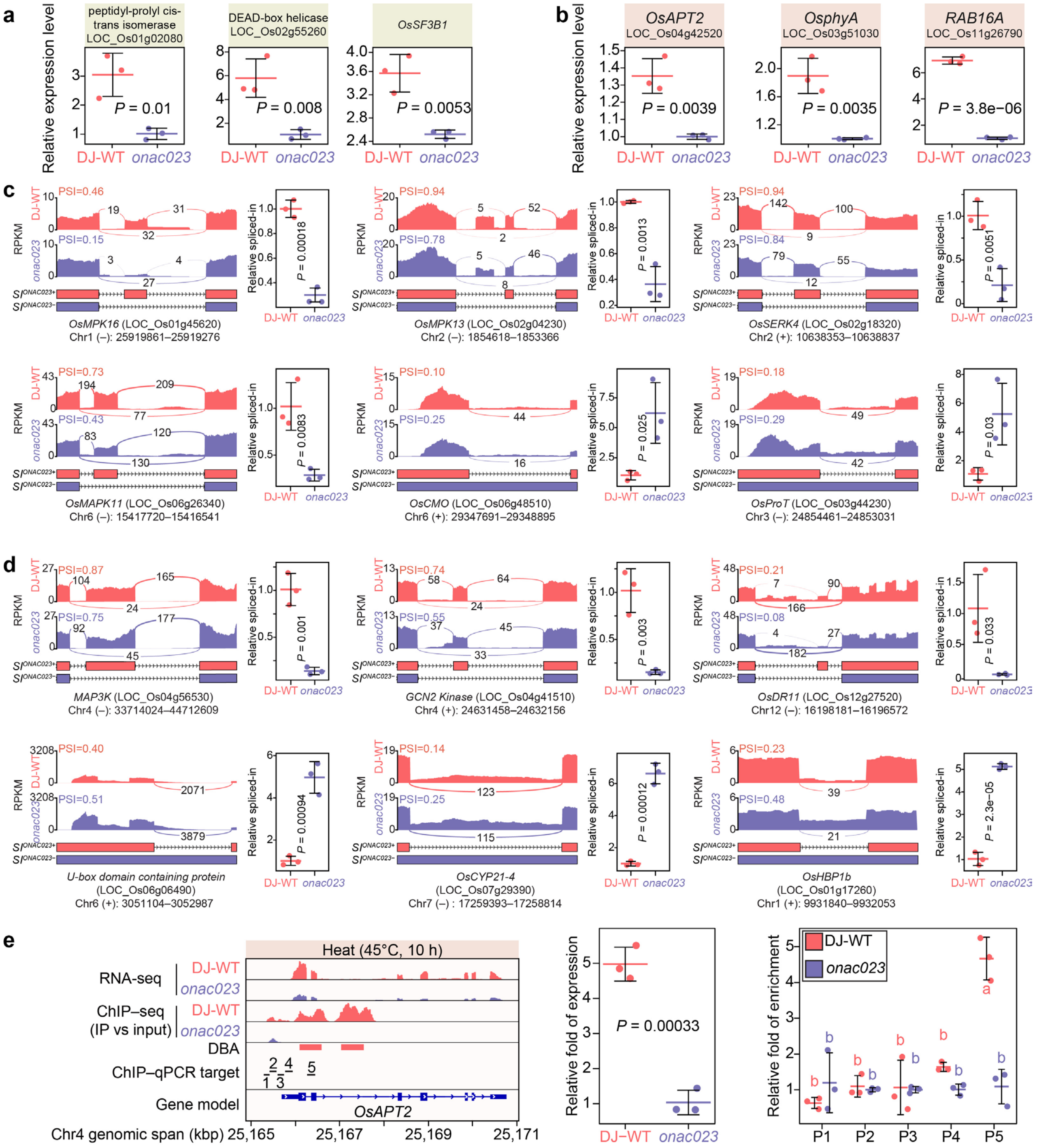

Supplementary Fig. 20: Additional qPCR validation of ONAC023-TGs and DASGs (supports Fig. 8 and 9).

a RT–qPCR validation on selected ONAC023-TGs (LOC_Os01g02080 and LOC_Os02g55260) and DEG (*OsSF3B1*) under drought stress. **b** RT–qPCR validation on the selected ONAC023-TG (*RAB16A*) and the DEGs (*OsAPT2* and *OsphyA*) under drought stress. **c**–**d** RT–qPCR validation

on the ASE within selected DASGs under drought (**c**) or heat (**d**) stress. Sashimi plots in **c** and **d** were generated for the corresponding ASE regions to exhibit the RNA-seq reads densities and the average junction reads numbers. Cross-bars indicate mean ± SD of three repeats of the treatments. *P*-values were determined by two-tailed Student's *t*-test. **e** Validation of ONAC023-targeting at *OsAPT2* in heat stress-treated (43°C, 4 h) inflorescence by RT–qPCR and ChIP–qPCR. The amplified regions of ChIP–qPCR assay are indicated in the genome tracks of the 45°C-treated seedlings. Cross-bars indicate mean ± SD of the relative enrichment (IP vs input, for ChIP–qPCR) or relative fold of expression (for RT–qPCR) based on three independent replicates. Significances ($P < 0.05$) were determined by two-tailed Student's *t*-test (for RT–qPCR) and by one-way ANOVA–HSD test (two-sided, for ChIP–qPCR), respectively. Exact *P*-values and details of the one-way ANOVA–HSD test can be found in the Source Data file. Source data are provided as a Source Data file.

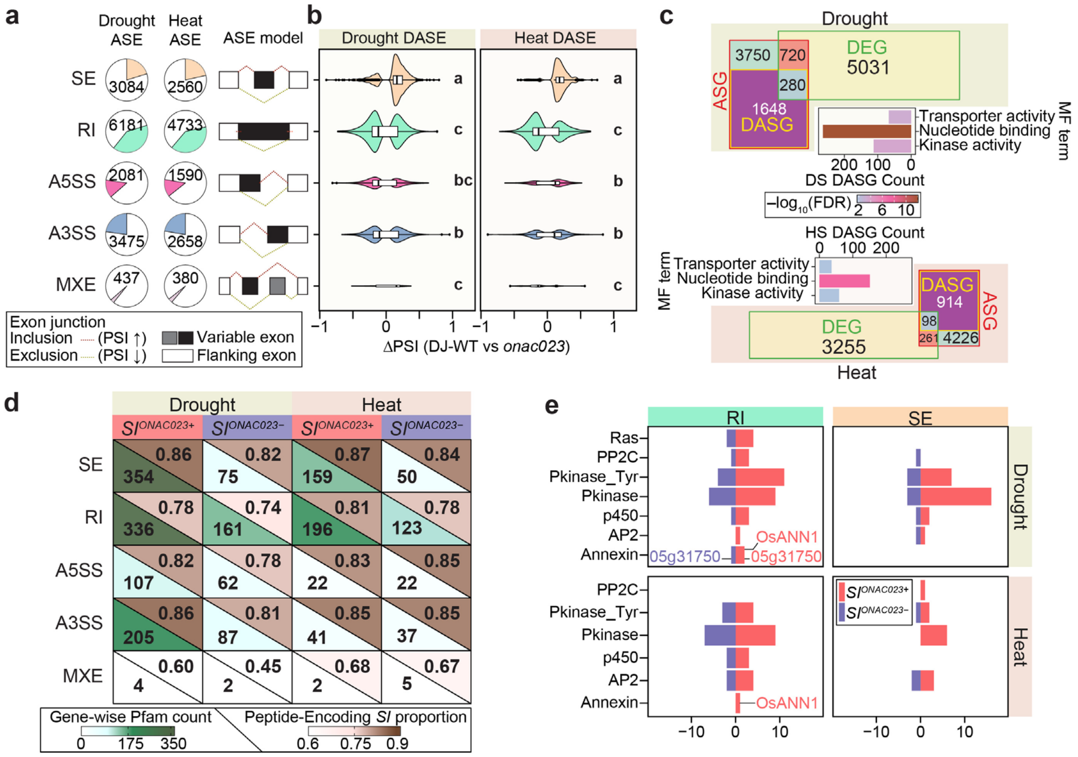

Supplementary Fig. 21: ONAC023 mediates the regulations on alternative splicing under drought and heat stresses (supports Fig. 9).

a Overview on the AS in DJ-WT and *onac023* under drought and heat stresses. Proportions of the five AS types under either treatment are shown in the pie charts. RI, retained intron; SE, skipped exon; A5SS, alternative 5′ splice site; A3SS, alternative 3′ splice site; MXE, mutually exclusive exon. **b** DASE between DJ and *onac023* are illustrated in the violin plot in terms of their ΔPSI values. Letter symbols denote significances ($P < 0.05$) determined by one-way ANOVA–HSD test (two-sided) **c** Square venn diagrams indicating the number and relations of ASGs, DASGs, and DEGs under each treatment. Categories are distinguished by the outline color. The histograms show the GO molecular function (MF) enrichment analysis on the DASGs under drought and heat stresses. Length of the histogram represents the enriched DASG numbers by the MF terms, and the color represent the significance (by hypergeometric test with Benjamini-Hochberg adjustment) of the enrichment. **d** Comprehensive heatmap illustrating the proportion of the peptide-encoding isoforms in the $SI^{ONAC023+}$ or $SI^{ONAC023-}$ (the upper-right value in each cell) and the number of gene-wise Pfam annotations based on the encoded peptide by $SI^{ONAC023+}$ or $SI^{ONAC023-}$ (the bottom-left

value in each cell). **e** Comparative view of the representative gene-wise Pfam categories of the RI and SE-involving *SI*$^{ONAC023+}$ or *SI*$^{ONAC023–}$ under the drought and heat stresses. Length of the histogram represents the number of gene-wise Pfam annotations. Annotated annexin is labeled as an example by the gene name or MSU v7.0 accession number. Source data are provided as a Source Data file.

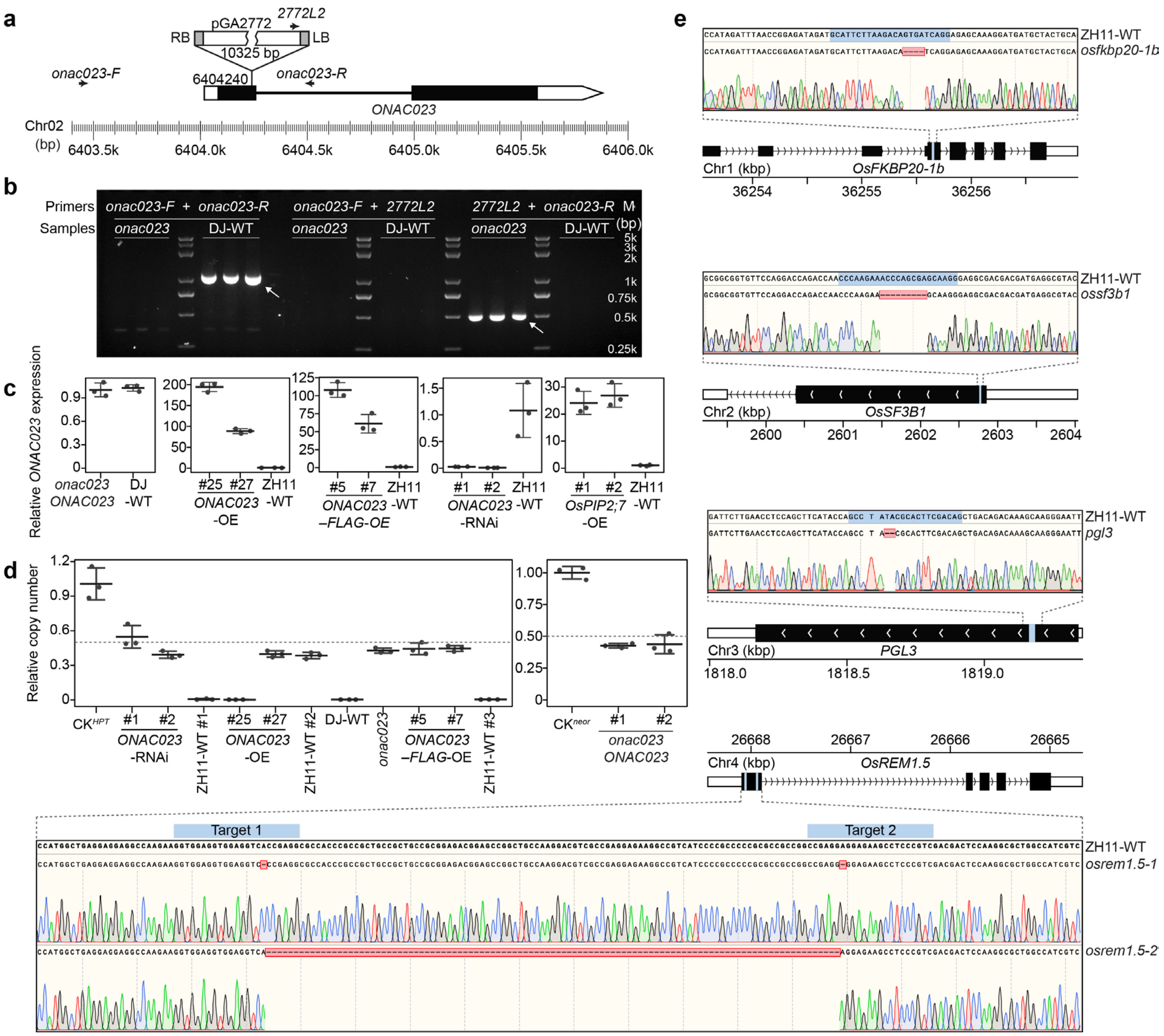

Supplementary Fig. 22: Genotype validation of the transgenic lines in this study (supports Figs. 2, 3, and 8).

a The schematic of the T-DNA insertion in the *onac023* mutant. LB and RB, left and right boundaries of the T-DNA. **b** Genotype validation on the *onac023*. The primers are as shown in **a**. Three batches of samples (> 5 plants each) were examined. M, DNA marker. **c** Expression validation on the T_1 transgenic lines. For the *ONAC023*-OE and *ONAC023–FLAG*-OE lines, samples (at least 10 seedlings per replicate) were harvested after 6-h heat stress treatment; for the rest of the samples, a 7-d drought treatment was applied. **d** Copy number of transgenic events validated by qPCR using the T_0 transgenic lines. Single copy plants were determined if the relative

copy number of *HPT* (*Hygromycin B phosphotransferase*) or *neo*^{r} gene in the plants were about half of the abundance in the predefined homozygous single-copy control lines (CK^{HPT} and CK^{neor}, scaled as 2). **e** Genotype validations on the mutated sites in *osfkbp20-1b*. *ossf3b1*, and *pgl3*. The CRISPR-Cas9 targeting sites are covered by blue rectangles, and the mutated bases are marked by red rectangles. Cross-bars in **c** and **d** indicate mean ± SD (> 5 plants for each replicate). Source data are provided as a Source Data file.

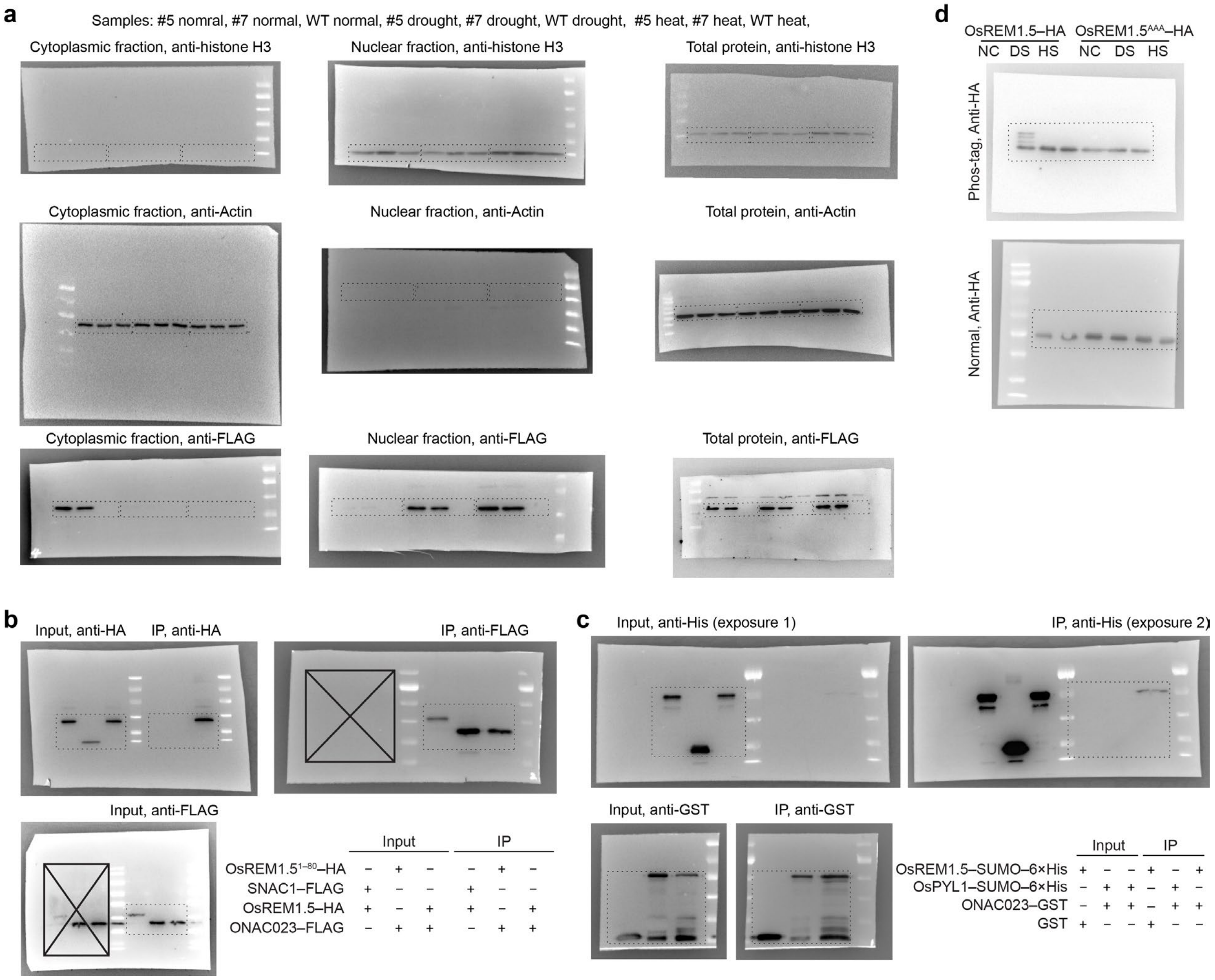

Supplementary Fig. 23: Uncropped images of the Western blot membranes (supports Figs. 4, 5, and 7).

a–**d** Uncropped images corresponding to Fig. 4b (**a**), Fig. 5b (**b**), Fig. 5c (**c**), and Fig. 7b (**d**). Lanes irrelevant to this study are marked with cross. The two images on top of panel **c** show the same membrane but with different exposures.
