# Supplementary material for: Stress-induced nuclear translocation of ONAC023 improves drought and heat tolerance through multiple processes in rice
Source: Nat Commun. 2024 Jul 13;15:5877. doi: 10.1038/s41467-024-50229-9 (PMC11245485; doi:10.1038/s41467-024-50229-9)
Supplement: Supplementary file 3 — Description of additional supplementary files [file 41467_2024_50229_MOESM3_ESM.pdf]

<u>Description of Additional Supplementary Files</u>

Supplementary Data 1. Rice cultivars and their image-traits used in the association study (supports Fig. 1).

Supplementary Data 2. Genetic data in the illustrated region in Fig. 1c (supports Fig. 1).

Supplementary Data 3. List of putative ONAC023-interacting proteins identified by IP-MS assay under drought and heat stresses (supports Fig. 5).

Supplementary Data 4. Unified gene models used for the high-throughput sequencing analysis in this study (supports Fig. 8).

Supplementary Data 5. List of DJ-WT vs *onac023* DEGs under drought and heat stresses (supports Fig. 8).

Supplementary Data 6. List of the ONAC023-TGs (supports Fig. 8).

Supplementary Data 7. GO enrichment results on the ONAC023-TGs (supports Fig. 8).

Supplementary Data 8. List of DJ-WT vs onac023 DASGs (supports Fig. 9).

Supplementary Data 9. GO enrichment results on the DASGs (supports Fig. 9).

Supplementary Data 10. List of the gene-wise Pfam annotations under drought and heat stresses (supports Fig. 9).

Supplementary Data 11. Primers used in this study (supports Figs. 1–9).
