# Supplementary material for: Stress-induced nuclear translocation of ONAC023 improves drought and heat tolerance through multiple processes in rice
Source: Nat Commun. 2024 Jul 13;15:5877. doi: 10.1038/s41467-024-50229-9 (PMC11245485; doi:10.1038/s41467-024-50229-9)
Supplement: Supplementary file 15 — Reporting Summary [file 41467_2024_50229_MOESM15_ESM.pdf]

nature portfolio

Corresponding author(s): Yujie Fang and Lizhong Xiong

Last updated by author(s): Jun 15, 2024

Reporting Summary

Nature Portfolio wishes to improve the reproducibility of the work that we publish. This form provides structure for consistency and transparency in reporting. For further information on Nature Portfolio policies, see our Editorial Policies and the Editorial Policy Checklist.

Statistics

For all statistical analyses, confirm that the following items are present in the figure legend, table legend, main text, or Methods section.

n/a	Confirmed	
☐	☒	The exact sample size (*n*) for each experimental group/condition, given as a discrete number and unit of measurement
☐	☒	A statement on whether measurements were taken from distinct samples or whether the same sample was measured repeatedly
☐	☒	The statistical test(s) used AND whether they are one- or two-sided<br>*Only common tests should be described solely by name; describe more complex techniques in the Methods section.*
☒	☐	A description of all covariates tested
☐	☒	A description of any assumptions or corrections, such as tests of normality and adjustment for multiple comparisons
☐	☒	A full description of the statistical parameters including central tendency (e.g. means) or other basic estimates (e.g. regression coefficient) AND variation (e.g. standard deviation) or associated estimates of uncertainty (e.g. confidence intervals)
☐	☒	For null hypothesis testing, the test statistic (e.g. *F*, *t*, *r*) with confidence intervals, effect sizes, degrees of freedom and *P* value noted<br>*Give P values as exact values whenever suitable.*
☒	☐	For Bayesian analysis, information on the choice of priors and Markov chain Monte Carlo settings
☒	☐	For hierarchical and complex designs, identification of the appropriate level for tests and full reporting of outcomes
☐	☒	Estimates of effect sizes (e.g. Cohen's *d*, Pearson's *r*), indicating how they were calculated

Our web collection on statistics for biologists contains articles on many of the points above.

Software and code

Policy information about availability of computer code

Data collection

No software was used in data collection.

Data analysis

The following open-source software were used in data analysis:
Fastp v0.23.2 (https://github.com/OpenGene/fastp)
Bowtie2 v2.4.5 (https://bowtie-bio.sourceforge.net/bowtie2/index.shtml)
MACS2 v2.2.7.1 (https://github.com/macs3-project/MACS)
Deeptools v3.5.1 (https://github.com/deeptools/deepTools)
R v4.1.3 (https://www.r-project.org/)
Cytoscape v3.9.0 (https://cytoscape.org/)
ImageJ v2.3.0 (https://imagej.net/ij/download.html)
HMMER v3.3.2 (http://hmmer.org/)
SATR v2.52b (https://github.com/alexdobin/STAR)
StringTie v1.3.1 (https://ccb.jhu.edu/software/stringtie/)
CPC2 v2.0 (http://cpc2.gao-lab.org/)
rMATS v4.1.2 (https://github.com/Xinglab/rmats-turbo)
Tassel 5 v5.2.72 (https://tassel.bitbucket.io)
VCFtools v0.1.16 (https://vcftools.github.io/)
IGV v2.16.0 (https://igv.org/)

For manuscripts utilizing custom algorithms or software that are central to the research but not yet described in published literature, software must be made available to editors and reviewers. We strongly encourage code deposition in a community repository (e.g. GitHub). See the Nature Portfolio guidelines for submitting code & software for further information.

Data

Policy information about availability of data

All manuscripts must include a data availability statement. This statement should provide the following information, where applicable:

- Accession codes, unique identifiers, or web links for publicly available datasets
- A description of any restrictions on data availability
- For clinical datasets or third party data, please ensure that the statement adheres to our policy

The raw sequencing reads as well as the processed data files generated during this study were deposited into the National Center for Biotechnology Information (NCBI) Gene Expression Omnibus (GEO) under the accession code GSE183241 [https://www.ncbi.nlm.nih.gov/geo/query/acc.cgi?acc=GSE183241]. The IP-MS data have been deposited to the ProteomeXchange Consortium via the PRIDE partner repository with the dataset identifier PXD046729 [http://www.ebi.ac.uk/pride/archive/projects/PXD046729]. The public phosphoproteome data of drought-stress-treated HY73 (Hanyou73, Oryza sativa ssp. indica) can be found in ProteomeXchange under the accession code PXD031505 [https://proteomecentral.proteomexchange.org/cgi/GetDataset?ID=PXD031505]. The minimum dataset required to reproduce the figures in this article have been deposited to the Figshare database83 [https://doi.org/10.6084/m9.figshare.26039821]. Sequence data of genes mentioned in this article can be found in the Rice Genome Annotation Project or EMBL data libraries under the following accession numbers: ONAC023 (LOC_Os02g12310) [http://rice.uga.edu/cgi-bin/ORF_infopage.cgi?orf=LOC_Os02g12310.1], OsREM1.5 (LOC_Os04g45070) [http://rice.uga.edu/cgi-bin/ORF_infopage.cgi?orf=LOC_Os04g45070.1], OsIMP-α1a (LOC_Os01g14950) [http://rice.uga.edu/cgi-bin/ORF_infopage.cgi?orf=LOC_Os01g14950.1], OsIMP-α1b (LOC_Os05g06350) [http://rice.uga.edu/cgi-bin/ORF_infopage.cgi?orf=LOC_Os05g06350.1], SNAC1 (LOC_Os03g60080) [http://rice.uga.edu/cgi-bin/ORF_infopage.cgi?orf=LOC_Os03g60080.1], OsPYL1 (LOC_Os10g42280) [http://rice.uga.edu/cgi-bin/ORF_infopage.cgi?orf=LOC_Os10g42280.1], OsFKBP20-1b (LOC_Os01g62610) [http://rice.uga.edu/cgi-bin/ORF_infopage.cgi?orf=LOC_Os01g62610.1], OsSF3B1 (LOC_Os02g05410) [http://rice.uga.edu/cgi-bin/ORF_infopage.cgi?orf=LOC_Os02g05410.1], PGL3 (LOC_Os03g03990) [http://rice.uga.edu/cgi-bin/ORF_infopage.cgi?orf=LOC_Os03g03990.1], OsPIP2;7 (LOC_Os09g36930) [http://rice.uga.edu/cgi-bin/ORF_infopage.cgi?orf=LOC_Os09g36930.1], OsANN1 (LOC_Os02g51750) [http://rice.uga.edu/cgi-bin/ORF_infopage.cgi?orf=LOC_Os02g51750.1], AtREM1.3 (AT2G45820) [https://plants.ensembl.org/Arabidopsis_thaliana/Gene/Summary?g=AT2G45820;r=2:18862953-18864741;t=AT2G45820.1], SPS (LOC_Os01g69030) [http://rice.uga.edu/cgi-bin/ORF_infopage.cgi?orf=LOC_Os01g69030.1], StREM1.3 (DN921712) [https://www.ebi.ac.uk/ena/browser/view/DN921712], Ubiquitin (LOC_Os03g13170) [http://rice.uga.edu/cgi-bin/ORF_infopage.cgi?orf=LOC_Os03g13170.1]. Source data are provided with this paper.

Research involving human participants, their data, or biological material

Policy information about studies with human participants or human data. See also policy information about sex, gender (identity/presentation), and sexual orientation and race, ethnicity and racism.

Reporting on sex and gender	n/a
Reporting on race, ethnicity, or other socially relevant groupings	n/a
Population characteristics	n/a
Recruitment	n/a
Ethics oversight	n/a

Note that full information on the approval of the study protocol must also be provided in the manuscript.

Field-specific reporting

Please select the one below that is the best fit for your research. If you are not sure, read the appropriate sections before making your selection.

☒ Life sciences ☐ Behavioural & social sciences ☐ Ecological, evolutionary & environmental sciences

For a reference copy of the document with all sections, see nature.com/documents/nr-reporting-summary-flat.pdf

Life sciences study design

All studies must disclose on these points even when the disclosure is negative.

Sample size

Required experimental sample sizes were determined based on our previous studies on rice functional genes (Tang et al., 2016; Shen et al., 2017; Yang et al., 2020).

Ref:

Shen J, Liu J, Xie K, Xing F, Xiong F, Xiao J, Li X, and Xiong L (2017). Translational repression by a miniature inverted-repeat transposable element in the 3' untranslated region. Nat Commun 8: 14651

Tang N, Ma S, Zong W, Yang N, Lv Y, Yan C, Guo Z, Li J, Li X, Xiang Y, et al. (2016). MODD mediates deactivation and degradation of OsbZIP46 to negatively regulate ABA signaling and drought resistance in rice. Plant Cell 28: 2161-2177

Yang J, Chang Y, Qin Y, Chen D, Zhu T, Peng K, Wang H, Tang N, Li X, Wang Y, et al. (2020). A lamin-like protein OsNMCP1 regulates drought resistance and root growth through chromatin accessibility modulation by interacting with a chromatin remodeller OsSWI3C in rice. New Phytol 227: 65-83

Data exclusions | No data was excluded from our analysis.

Replication | All experiments were repeated at least three times, and we have stated the number of replications in the corresponding figure legends.

Randomization | For each experiment, seedlings and protoplasts were selected, and the pots for the treatment groups and control groups were randomly placed. For in-field studies, all rice plants were planted in randomized blocks.

Blinding | Data collection and analysis were performed in a blinded manner.

Reporting for specific materials, systems and methods

We require information from authors about some types of materials, experimental systems and methods used in many studies. Here, indicate whether each material, system or method listed is relevant to your study. If you are not sure if a list item applies to your research, read the appropriate section before selecting a response.

Materials & experimental systems

n/a	Involved in the study
☐	☒ Antibodies
☒	☐ Eukaryotic cell lines
☒	☐ Palaeontology and archaeology
☒	☐ Animals and other organisms
☒	☐ Clinical data
☒	☐ Dual use research of concern
☐	☒ Plants

Methods

n/a	Involved in the study
☐	☒ ChIP-seq
☒	☐ Flow cytometry
☒	☐ MRI-based neuroimaging

Antibodies

Antibodies used

The commercialized antibodies were ordered from the Sigma-Aldrich and Abclonal companies. The catalog number and the specific information are listed below: anti-FLAG antibody (Sigma-Aldrich, F3165, 1:5000 dilution), anti-Actin antibody (ABclonal, AC009, 1:5000 dilution), anti-histone H3 antibody (ABclonal, A2348, 1:5000 dilution), anti-HA antibody (ABclonal, AE008, 1:2500 dilution), anti-His antibody (ABclonal, AE003, 1:3000 dilution), anti-GTS antibody (ABclonal, AE001, 1:5000 dilution), and Alexa Fluor 594-conjugated secondary antibody (ABclonal, AS054).
The specific antibody against ONAC023 protein (anti-ONAC023 polyclonal antibody) was produced by Abmart company with a catalog number 8114-1hz.

Validation

The ONAC023-specific antibody was validated by Western blot including DJ-WT, onac023 mutant, and the transgenic complementary plants. And its specificity was double checked using the anti-FLAG antibody in the ONAC023-FLGA-OE plants (see Fig. S11.
The specificities of the commercialized antibodies can be found in the manufacturers' websites, listed as follows:
anti-FLAG antibody (Sigma-Aldrich, F3165): https://www.sigmaaldrich.cn/CN/zh/product/sigma/f3165
anti-Actin antibody (ABclonal, AC009): https://abclonal.com.cn/catalog/AC009
anti-histone H3 antibody (ABclonal, A2348): https://abclonal.com.cn/catalog/A2348
anti-HA antibody (ABclonal, AE008): https://abclonal.com.cn/catalog/AE008
anti-His antibody (ABclonal, AE003): https://abclonal.com.cn/catalog/AE003
anti-GTS antibody (ABclonal, AE001): https://abclonal.com.cn/catalog/AE001
Alexa Fluor 594-conjugated secondary antibody (ABclonal, AS054): https://abclonal.com.cn/catalog/AS054

Plants

Seed stocks

The CRISPR-Cas9-mediated mutants of the ONAC023-TGs were generated by BIOGEL GeneTech Co., Ltd (Wuhan, China) with the accession numbers #1702-26560 (osfkbp20-1b), #1702-26562 (ossf3b1), and #1702-26564 (pgl3). The DJ background T-DNA insertion mutant onac023 (accession ID: 2D-30113) was obtained from Postech (S. Korea, Plant Functional Genomics Laboratory).
Seeds of the other transgenic plants generated by our lab are available upon request.

Novel plant genotypes

To create the OE constructs, the coding sequence (CDS) of ONAC023 or OsPIP2;7 was amplified and inserted into the pU1301 backbone, a modified version of pCAMBIA1301 with the insertion of a maize polyubiquitin-1 promoter and a nopaline synthase polyadenylation signal (NOS) terminator (Qiu et al., 2007). The ONAC023 CDS tagged with a 3× FLAG sequence at the 3′ end was integrated into pU1301 to generate the ONAC023–FLAG-OE construct. The ONAC023-RNAi construct was generated by insertion of the palindromic sequence of a partial ONAC023 CDS fragment into the pDS1301 vector (Chu et al., 2006). The above constructs were introduced into ZH11 calli through Agrobacterium-mediated transformation (Hiei et al., 1994). To generate the complementary construct, the gene body and the 3500-bp upstream genomic sequence of ONAC023 were amplified from DJ genomic DNA and integrated into the pCAMBIA2301 backbone, and the construct was subsequently used to transfect the onac023-derived calli. The CRISPR-Cas9-mediated mutation construct of OsREM1.5 was generated by integrating spacer sequences referring to two target sites in OsREM1.5 coding region into the pRGEB32 vector as described previously (Xie et al., 2015). Primers used in this study are listed in Supplementary Data 12. Details of each transgenic line are as follows:
For ONAC023-OE transgenic lines, there are 33 independent transformation events in T0 generation, and two lines with single-copy T-DNA insertion were isolated and characterized. For ONAC023–FLAG-OE transgenic lines, there are 10 independent transformation events in T0 generation, and two lines with single-copy T-DNA insertion were isolated and characterized. For onac023 ONAC023 transgenic lines, there are 8 independent transformation events in T0 generation, and two lines with single-copy (in addition to the T-DNA at ONAC023 locus) T-DNA insertion were isolated and characterized. For OsPIP2;7-OE transgenic lines, there are 22 independent transformation events in T0 generation, and two lines with single-copy T-DNA insertion were isolated and characterized (at T1 generation). T3 (or higher) generation transgenic lines with stable expression level and phenotype were used in the experiments in this article.
For ONAC023-RNAi transgenic lines, there are 28 independent transformation events in T0 generation, and two lines with single-copy

Authentication

The transcript abundance of the OE lines was detected by RT–qPCR and the copy numbers were analyzed by genomic DNA-based qPCR using the SPS gene as an internal reference as described previously (Yang et al., 2005; Chang et al., 2017) (see Fig. S20). The T-DNA insertion mutant was validated by PCR amplification of the insertion region and DNA-based qPCR analysis of the insertion number. The CRISPR-Cas9-mediated mutants were validated by PCR coupled with sequencing. Targeting sites of the CRISPR-Cas9 were positioned in genomic regions with low- homogeneity and the experiments involving these mutants were performed using the derived WT plants as control to minimize potential off-target effect.
Ref:
Chang Y, Nguyen BH, Xie Y, Xiao B, Tang N, Zhu W, Mou T, and Xiong L (2017). Co-overexpression of the constitutively active form of OsbZIP46 and ABA-activated protein kinase SAPK6 improves drought and temperature stress resistance in rice. Front Plant Sci 8: 1102
Yang LT, Ding JY, Zhang CM, Jia JW, Weng HB, Liu WX, and Zhang DB (2005). Estimating the copy number of transgenes in transformed rice by real-time quantitative PCR. Plant Cell Rep 23: 759-763.

ChIP-seq

Data deposition

☒ Confirm that both raw and final processed data have been deposited in a public database such as GEO.

☒ Confirm that you have deposited or provided access to graph files (e.g. BED files) for the called peaks.

Data access links<br>*May remain private before publication.*	https://www.ncbi.nlm.nih.gov/geo/query/acc.cgi?acc=GSE183241
Files in database submission	RAW files:<br>GSM5553815 onac023_DS_IP_rep1_ChIPseq<br>GSM5553816 onac023_DS_IP_rep2_ChIPseq<br>GSM5553817 DJ_DS_IP_rep1_ChIPseq<br>GSM5553818 DJ_DS_IP_rep2_ChIPseq<br>GSM5553819 onac023_HS_IP_rep1_ChIPseq<br>GSM5553820 onac023_HS_IP_rep2_ChIPseq<br>GSM5553821 onac023_HS_IP_rep3_ChIPseq<br>GSM5553822 DJ_HS_IP_rep1_ChIPseq<br>GSM5553823 DJ_HS_IP_rep2_ChIPseq<br>GSM5553824 DJ_HS_IP_rep3_ChIPseq<br>GSM5553825 mutCK_ChIPseq_input<br>GSM5553826 onac023_input_ChIPseq<br>GSM6226382 ONAC023-FLAG_OE_5_NC_IP_rep1_ChIPseq<br>GSM6226383 ONAC023-FLAG_OE_5_NC_IP_rep2_ChIPseq<br>GSM6226384 ZH11-WT_NC_IP_rep1_ChIPseq<br>GSM6226385 ZH11-WT_NC_IP_rep2_ChIPseq<br>GSM6226386 ONAC023-FLAG_OE_5_NC_input_ChIPseq<br>GSM6226387 ZH11-WT_NC_input_ChIPseq<br><br>Processed data files:<br>GSE183241_ONAC023-FLAG_OE_5_NC_rep1_IP_vs_input.bw<br>GSE183241_ONAC023-FLAG_OE_5_NC_rep2_IP_vs_input.bw<br>GSE183241_ZH11-WT_rep1_IP_vs_input.bw<br>GSE183241_ZH11-WT_rep2_IP_vs_input.bw<br>GSE183241_mutCK_D.pooled.IP_vs_input.bw<br>GSE183241_mutCK_H.pooled.IP_vs_input.bw<br>GSE183241_mut_D.pooled.IP_vs_input.bw<br>GSE183241_mut_H.pooled.IP_vs_input.bw
Genome browser session<br>(e.g. UCSC)	n/a

Methodology

Replicates	2-3 replicates (with independent batches of seedlings and treatments) with high reproducibility (Pearson correlation > 0.8) were involved.
Sequencing depth	The sequencing depth of the samples ranged from 25-38, see Fig. S16.
Antibodies	The specific antibody against ONAC023 protein (anti-ONAC023 polyclonal antibody) was produced by Abmart company with a catalog number 8114-1hz. Anti-FLAG antibody was manufactured by Sigma-Aldrich (F3165).
Peak calling parameters	Peak calling was performed on nonredundant, uniquely mapped reads by MACS2 with parameters "--call-summits -f BAMPE -g 3.74e8 --mfold 2 50"
Data quality	Only peaks detected in at least two replicates were preserved to ensure quality. 1695 and 14290 candidate ONAC023-binding peaks were detected under drought and heat stress, respectively, under the threshold of FDR < 0.05 and FC >5.
Software	The raw sequencing reads were filtered by fastp (with the parameter "-q 30")(Chen et al., 2018) and mapped to rice genome assembly MSU v7.0 by bowtie2(Langmead and Salzberg, 2012) with parameters "--sensitive, --no-unal, -X 1000". Peak calling was performed on nonredundant, uniquely mapped reads by MACS2(Zhang et al., 2008). DBA (differential-binding area) analysis was performed by R package DiffBind (Ross-Innes et al., 2012) with its built-in DESeq2 analyzer.<br><br>Ref<br>Chen S, Zhou Y, Chen Y, and Gu J (2018). fastp: an ultra-fast all-in-one FASTQ preprocessor. Bioinformatics 34: i884-i890<br>Langmead B, and Salzberg SL (2012). Fast gapped-read alignment with Bowtie 2. Nat Methods 9: 357-359<br>Ross-Innes CS, Stark R, Teschendorff AE, Holmes KA, Ali HR, Dunning MJ, Brown GD, Gojis O, Ellis IO, Green AR, et al. (2012). Differential oestrogen receptor binding is associated with clinical outcome in breast cancer. Nature 481: 389-393<br>Zhang Y, Liu T, Meyer CA, Eeckhoute J, Johnson DS, Bernstein BE, Nusbaum C, Myers RM, Brown M, Li W, et al. (2008). Model-based analysis of ChIP-Seq (MACS). Genome Biol 9: R137
